# Telemedicine Technologies for Diabetes in Pregnancy: A Systematic Review and Meta-Analysis

**DOI:** 10.2196/jmir.6556

**Published:** 2016-11-09

**Authors:** Wai-Kit Ming, Lucy H Mackillop, Andrew J Farmer, Lise Loerup, Katy Bartlett, Jonathan C Levy, Lionel Tarassenko, Carmelo Velardo, Yvonne Kenworthy, Jane E Hirst

**Affiliations:** ^1^ Nuffield Department of Obstetrics & Gynaecology John Radcliffe Hospital University of Oxford United Kingdom; ^2^ Department of Obstetrics & GynaecologyThe First Affiliated Hospital Sun Yat-sen University Guangzhou China; ^3^ Women’s Centre Oxford University Hospitals NHS Trust Oxford United Kingdom; ^4^ Nuffield Department of Primary Care Health Sciences University of Oxford Oxford United Kingdom; ^5^ Institute of Biomedical Engineering Department of Engineering Science University of Oxford Oxford United Kingdom; ^6^ The Oxford Centre for Diabetes Endocrinology and Metabolism Oxford University Hospitals NHS Trust Oxford United Kingdom

**Keywords:** pregnancy, diabetes mellitus, telemedicine, review, meta-analysis, pregnancy in diabetics

## Abstract

**Background:**

Diabetes in pregnancy is a global problem. Technological innovations present exciting opportunities for novel approaches to improve clinical care delivery for gestational and other forms of diabetes in pregnancy.

**Objective:**

To perform an updated and comprehensive systematic review and meta-analysis of the literature to determine whether telemedicine solutions offer any advantages compared with the standard care for women with diabetes in pregnancy.

**Methods:**

The review was developed using the Preferred Reporting Items for Systematic Reviews and Meta-Analyses (PRISMA) framework. Randomized controlled trials (RCT) in women with diabetes in pregnancy that compared telemedicine blood glucose monitoring with the standard care were identified. Searches were performed in SCOPUS and PubMed, limited to English language publications between January 2000 and January 2016. Trials that met the eligibility criteria were scored for risk of bias using the Cochrane Collaborations Risk of Bias Tool. A meta-analysis was performed using Review Manager software version 5.3 (Nordic Cochrane Centre, Cochrane Collaboration).

**Results:**

A total of 7 trials were identified. Meta-analysis demonstrated a modest but statistically significant improvement in HbA1c associated with the use of a telemedicine technology. The mean HbA1c of women using telemedicine was 5.33% (SD 0.70) compared with 5.45% (SD 0.58) in the standard care group, representing a mean difference of −0.12% (95% CI −0.23% to −0.02%). When this comparison was limited to women with gestational diabetes mellitus (GDM) only, the mean HbA1c of women using telemedicine was 5.22% (SD 0.70) compared with 5.37% (SD 0.61) in the standard care group, mean difference −0.14% (95% CI −0.25% to −0.04%). There were no differences in other maternal and neonatal outcomes reported.

**Conclusions:**

There is currently insufficient evidence that telemedicine technology is superior to standard care for women with diabetes in pregnancy; however, there was no evidence of harm. No trials were identified that assessed patient satisfaction or cost of care delivery, and it may be in these areas where these technologies may be found most valuable.

## Introduction

Diabetes in pregnancy is a global problem and innovative solutions are required to prevent adverse outcomes in the mother and the offspring [1]. The prevalence of gestational diabetes mellitus (GDM) has increased dramatically with the International Diabetes Federation estimating that 1 in 7 pregnant women had GDM in 2015 [2,3]. The aims of clinical management, whether for women with type 1, type 2, or GDM, are to normalize maternal blood glucose to reduce complications and improve maternal and pregnancy outcomes [4]. Current evidence supports regular self-blood glucose monitoring (SBGM) up to 7 times a day, dietary and lifestyle counselling, and, frequently, hypoglycemic medications with dose titration in response to glycemic control [1,5,6]. Adequacy of glycemic control is determined by reviewing SBGM results, traditionally recorded by the woman by hand in paper diaries. The frequent need for outpatient visits to review these results as pregnancy progresses places pressure on maternity and diabetic services and is an inconvenience for pregnant women and their families.

Technological innovations present exciting opportunities for novel approaches to improve clinical care delivery for women with diabetes in pregnancy. Telemedicine (also known as telehealth) is defined as the provision of health services at a distance using a range of technologies [7]. The World Health Organization recommends telemedicine systems should be introduced where there is demand from patients [8]. With 1 in 3 people on the planet predicted to own a mobile phone by the end of 2016 [9], there is great enthusiasm among both patients and health care professionals to harness digital technologies to improve human health. In line with this, the number and sophistication of apps developed specifically for women with diabetes in pregnancy has increased [10,11]. Digital technologies in this patient group have most commonly been used to record and transmit blood glucose readings to the clinical care team between outpatient visits. This can involve either synchronous (ie, real-time) or asynchronous interactions, facilitating 2-way communication between the clinical care team and the pregnant woman [12,13]. Examples of technologies to perform this task include mobile apps, short message service (SMS), automated telephone support systems, Web-based diaries and decision-support systems, and integrated systems combining multiple elements of digital communication technologies (eg, mobile apps supported by Web platforms) [10,14-21].

Despite this enthusiasm, the benefits of telemedicine in women with diabetes in pregnancy remain uncertain [22]. Before recommending routine use and scale-up, ideally, there should be some evidence of benefit, or, at least no evidence of harm, when compared with traditional models of care. In addition to clinical benefit, telemedicine may offer advantages over standard care through improved efficiency of health care delivery, better maternal satisfaction with care [23,24], and economic savings related to fewer clinical visits [25].

The field of telemedicine is rapidly changing. We aimed to perform an updated and comprehensive systematic review and meta-analysis of the literature to determine whether, in pregnant women with any form of diabetes, telemedicine solutions offer any advantages compared with standard care. Outcomes were considered with respect to (1) maternal glycemic control, (2) pregnancy complications, (3) maternal satisfaction, and (4) costs of care.

## Methods

### Study Design

A research protocol was developed according to the Preferred Reporting Items for Systematic Reviews and Meta-Analyses (PRISMA) framework [26].

#### Search Strategy

The search strategy was developed with the advice of a professional librarian, with searches performed in SCOPUS (Medline, EMBASE, and Compendex) and PubMed to identify all relevant publications published between January 2000 and January 2016. This date restriction was selected as it was thought any telemedicine systems reported prior to this time would not be comparable with contemporary technology.

#### Inclusion Criteria

For the purpose of this review, any pregnant woman with a diagnosis of GDM (according to any criteria) or with preexisting type 1 diabetes or type 2 diabetes was eligible for inclusion. For this paper, telemedicine was defined as any system to monitor blood glucose remotely utilizing either fixed-line phones, mobile phones, or Internet-based systems. Databases were searched using the keywords tele*, digital*, comput*, *phone*, mobile*, app*, remote*, PDA, web*, tech*, Internet*, automat*, video*, wireless, short messag*, SMS, ehealth and e-health combined with gestational diabetes, GDM, pregnan* diabetes, pregnan* DM, and pregnan* gly*. These terms were combined using Boolean operators. The full search strategy for the SCOPUS and PubMed database (Textbox 1) was complemented with another approach involving the review of reference lists of retrieved trials. We limited our search to RCT.

#### Exclusion Criteria

Trials were excluded if they were quasi- or non-randomized, conducted in women where pregnancy status was not clearly stated, or the comparator group was another digital technology (rather than standard care). For practical reasons, the search was limited to English language publications.

**Search terms used to identify articles related to telemedicine or related technology used in gestational diabetes.**

1. tele*

2. digital*

3. comput*

4. *phone*

5. mobile*

6. app

7. apps

8. remote*

9. PDA

10. web*

11. tech*

12. internet*

13. automat*

14. video*

15. wireless

16. short messag*

17. SMS

18. Ehealth

19. e-health

20. 1 or 2 or 3 or 4 or 5 or 6 or 7 or 8 or 9 or 10 or 11 or 12 or 13 or 14 or 15 or 16 or 17 or 18 or 19

21. gestational diabetes

22. GDM

23. Pregnan* diabetes

24. Pregnan* DM

25. Pregnan* gly*

26. woman DM

27. 21 or 22 or 23 or 24 or 25 or 26

28. 20 and 27

### Study Selection Process

One of the authors (W Ming) independently screened the titles and abstracts of identified citations for potential eligibility. Two authors (W Ming and J Hirst) independently examined the full-text articles of eligible papers and extracted information about the exposures and outcomes using a predefined data extraction table.

### Outcomes

The primary outcome was maternal glycemic control. Owing to the challenges in quantifying glycemic control and lack of consensus in measuring and reporting this outcome in pregnancy, we chose to define glycemic control with respect to mean blood glucose during pregnancy monitoring (total, fasting, or 1-h or 2-h post-prandial blood, expressed in mmol/L), and final recorded HbA1c in pregnancy (reported as both % and mmol/mol).

Secondary outcomes included insulin usage (ie, the final dose of insulin in units), mode of delivery (vaginal delivery or cesarean section), and the proportion of cases of shoulder dystocia at birth. As poor glycemic control in pregnancy is associated with increased fetal size, we also compared differences in fetal size as defined by mean birth weight, rates of macrosomia (defined as birth weight >4000 g), and the proportion of babies that were large for gestational age (LGA; defined as birth weight for gestational age and gender >90th percentile using local references). Neonatal outcomes were also assessed including the need for any neonatal intensive care unit (NICU) admission, preterm birth <37 completed weeks, and neonatal hypoglycemia (defined as hypoglycemia requiring medical treatment; Multimedia Appendix 1).

### Data Extraction and Quality Assessment

Information on trial design and data on the primary and secondary outcomes were extracted by 2 reviewers, independently, using a predesigned Excel spreadsheet. Each trial was scored for the risk of bias using the Cochrane Collaboration Risk of Bias Tool. A third reviewer was available if there was a difference in opinion in interpreting the risk of bias.

#### Data Synthesis and Analysis

Meta-analysis was performed using Review Manager software (Version 5.3). Given that different technologies were assessed and the definitions of diabetes and standard care varied between the trials, we anticipated a large amount of heterogeneity in the results. Therefore, we applied random effects models with the I^2^ statistic reported. I^2^ values >50% are considered to indicate substantial heterogeneity. Results are presented as the differences in risk ratios for binary outcomes and mean difference for continuous variables, with 95% CI. Results were stratified by the diabetes type if more than 1 trial was available. For outcomes reported in only 1 trial or unable to be combined across trials, a narrative synthesis was presented.

## Results

### Study Selection and Study Characteristics

**Figure 1:**
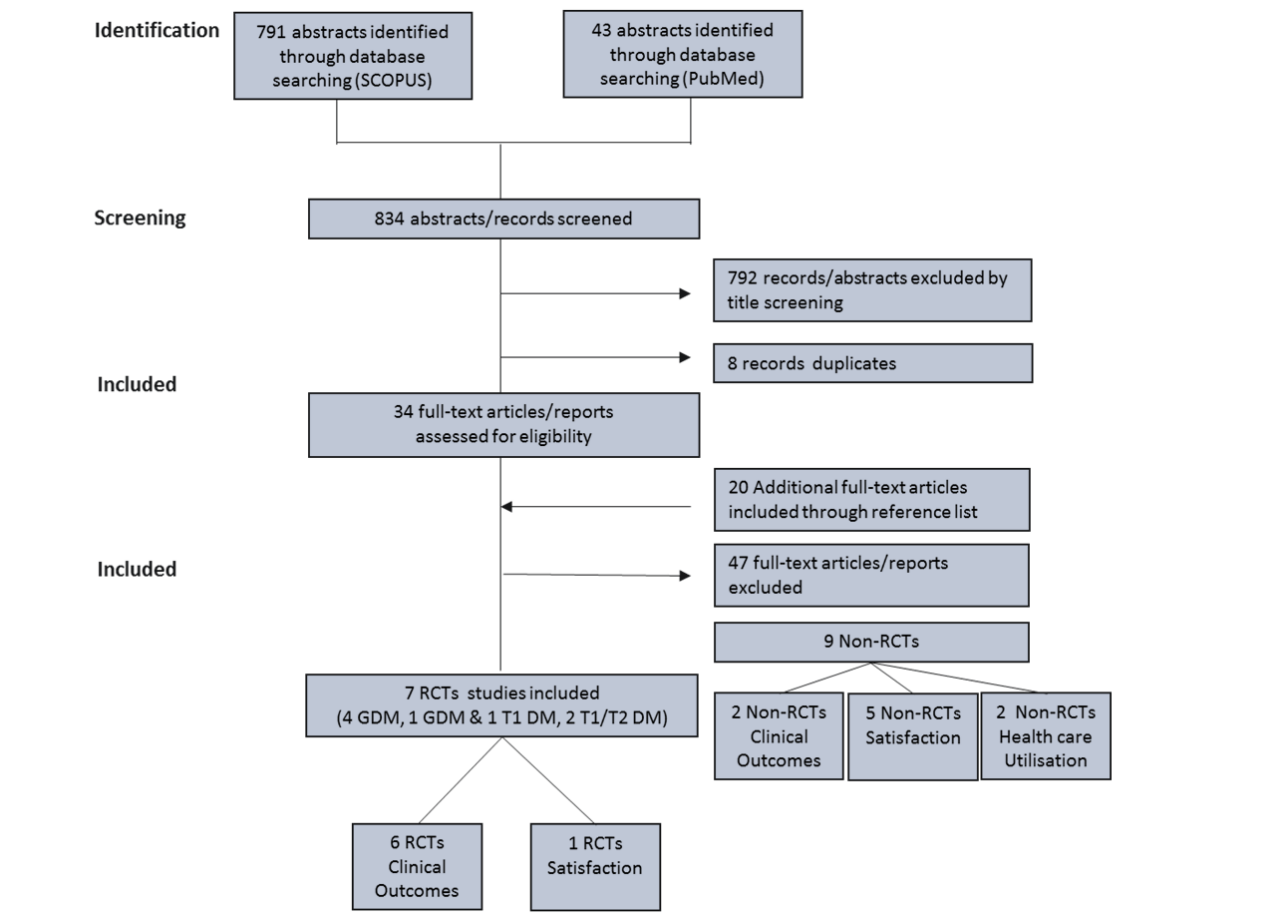
Study selection. RTC: randomized controlled trial; GDM: gestational diabetes mellitus; T1 DM: type 1 diabetes mellitus; T2 DM: type 2 diabetes mellitus.

The search and screening strategy is shown in Figure 1. Seven of the 54 trials selected for full-text review met the inclusion criteria, involving 579 women: 496 women with GDM (5 trials) [16,21,27-29] and 83 with type 1 diabetes (3 trials) [15,21,30]. The trial of Dalfra et al presented results separately for women with GDM and type 1 diabetes; thus, for analysis we present this trial stratified by diabetes type [21]. All trials were small in size, ranging from 19 to 203 women with a median of 57 (interquartile range 32-85). The 7 trials were all conducted in high-income countries (5 in Europe and 2 in North America). See Multimedia Appendix 1.

### Modes of Communication and Type of Intervention

Technologies assessed were modem transmission of blood glucose readings to a central hospital computer [15], websites accessible to patients and health care professionals [17,18], a telephone system that translated blood glucose readings into audio tones to transmit them to a computer database [21], SMS transmissions of blood glucose readings to a central database [19], and a telemedicine hub located in the woman’s home, which transmitted data every week to a clinical team through the Internet [16]. All trials described the comparison groups as receiving “routine care.” However, this ranged from information given only about the method of blood glucose monitoring (ie, paper log books), to detailed descriptions of care pathways. The frequency of clinic visits differed between the trials, ranging from weekly to monthly visits.

### Methodological Quality Assessment

**Figure 2:**
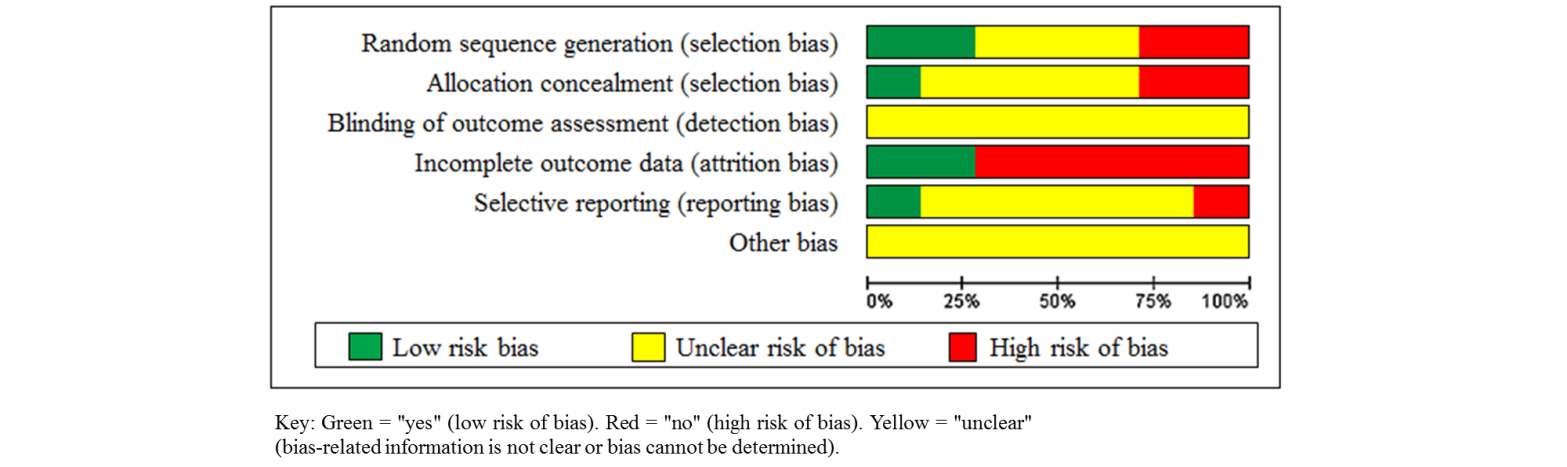
Distribution of bias in the included trials.

**Figure 3:**
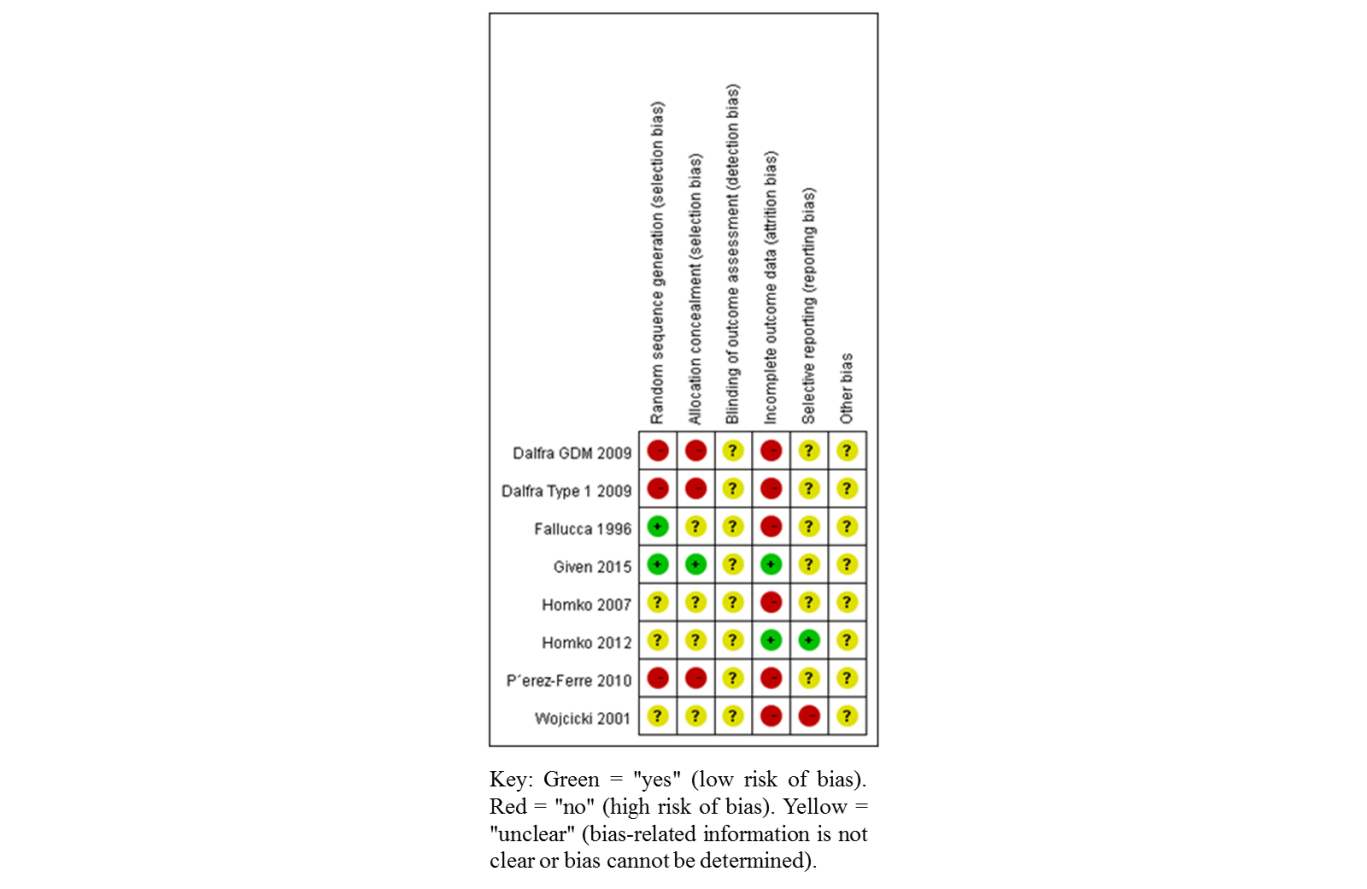
Risk of bias in the included trials.

Overall, all the trials displayed potential sources of methodological bias (Figures 2 and 3). Owing to the nature of the intervention, blinding of participants and health care providers was not possible and therefore we elected to not include this as part of the risk of bias assessment. Considering the method of randomization, 2 trials were found to be at low risk of bias, reporting the use of computerized stratified block randomization [15,16]. The remainder either used methods that were likely to be of high risk of bias, or did not report this component. Only 1 trial reported use of an adequate allocation concealment method [17]. Two trials gave a full description of participants and losses to follow-up during their trial [16,17]. Other trials reported losses to follow-up or postrandomization exclusions, which potentially may have affected the results. Reporting bias is the selective reporting of some outcomes but not others depending on the nature and direction of the results [31]. Only 1 included trial was judged to be at low risk of reporting bias [17], reporting a comprehensive range of glucose and clinical outcomes.

All the identified trials addressed clinical outcomes. Only 1 trial also reported maternal satisfaction; however, no comparative statistics were given between the intervention and the control groups. No trial presented any data on health economic outcomes.

### Maternal Glycemic Control

**Figure 4:**
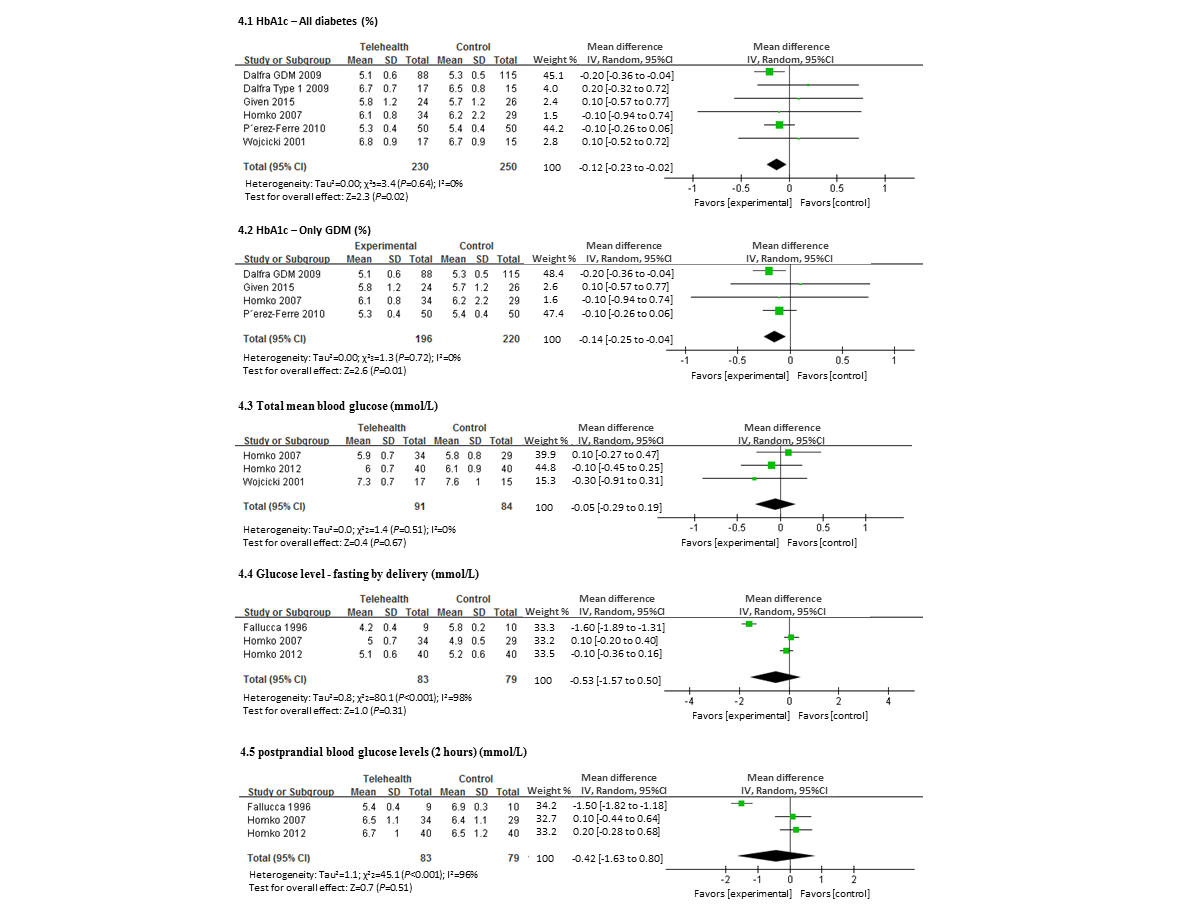
Forest plot showing the pooled HbA1c and blood glucose level (telemedicine vs control group).

HbA1c was the most commonly reported glycemic outcome in 5 trials [21,28-30]. Meta-analysis demonstrated a modest, but statistically significant, improvement in HbA1c associated with the use of a telemedicine. The mean HbA1c of women using telemedicine was 5.33% (SD 0.70) compared with 5.45% (SD 0.58) in the standard care group, representing a mean difference of −0.12% (95% CI −0.23% to −0.02%). When this comparison was limited to the 4 trials of women with GDM only, the difference was slightly greater [21,28,29]. The mean HbA1c of women with GDM using telemedicine was 5.23% (SD 0.70) compared with 5.37% (SD 0.61) in the standard care group, mean difference −0.14% (95% CI −0.25% to −0.04%). Three trials (175 women: 143 GDM and 32 type 1) compared the overall mean blood glucose levels between the intervention (telemedicine) and control (standard care) groups [27,28,30]. Meta-analysis of these trials demonstrated no evidence of difference in mean blood glucose levels; however, this was in keeping with the lack of difference in HbA1c also observed in these individual trials (Figure 4). Two of these trials reported differences between fasting and 2 h postprandial blood glucose, however, no significant difference was demonstrated between the groups [15,28]. One trial in women with type 1 diabetes reported the mean units of insulin used in each group [15]. For these 19 women, the telemedicine group used a greater total dose of insulin compared with standard care, 54 units (SD 7 units) and 36 units (SD 6 units), respectively.

### Maternal and Neonatal Clinical Outcomes

**Figure 5:**
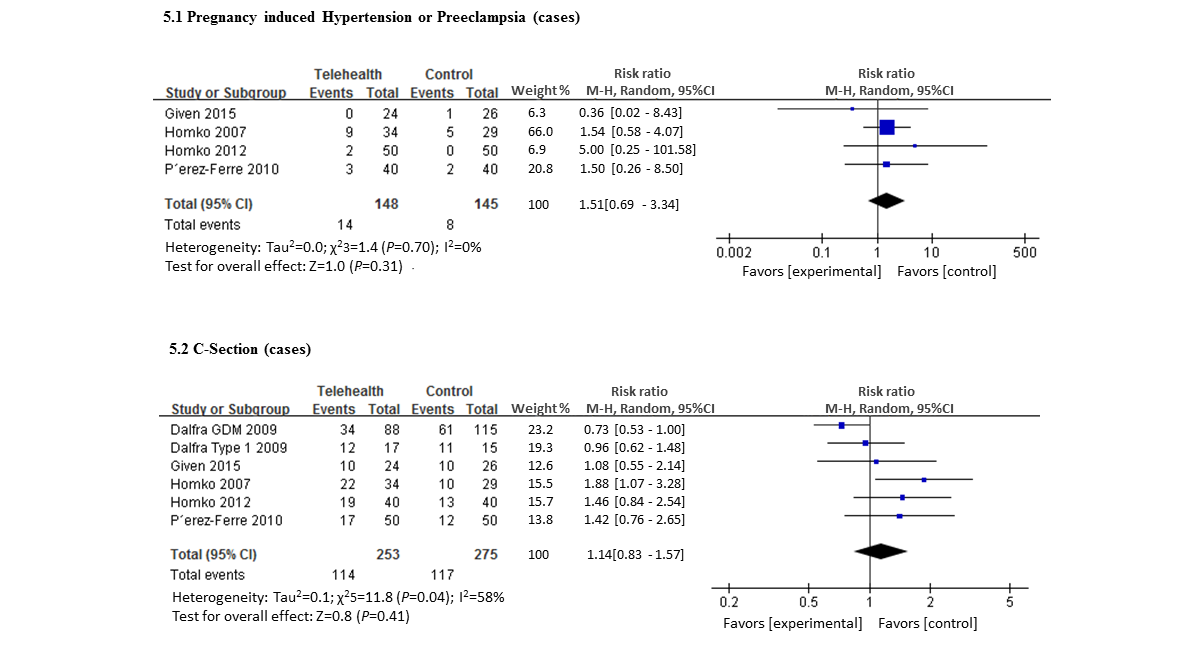
Forest plot showing the pooled clinical parameter—maternal outcomes (telemedicine vs control group).

Maternal outcomes were reported variously across the trials. A total of 4 trials (148 women using telemedicine and 145 controls) reported differences between rates of pregnancy-induced hypertension or preeclampsia [27-29]. In these trials, 7.5% of women overall had either of these conditions; however, there was no difference in the risk ratio between the telemedicine or control groups (Figure 5). When considering the mode of delivery, rates of Cesarean section were high in both the groups (50.0% in the telemedicine and 45.0% in the control) with no difference in the risk ratio. Only 2 trials (150 women) reported shoulder dystocia [29], however, with only 1 case of shoulder dystocia meta-analysis was not possible.

**Figure 6:**
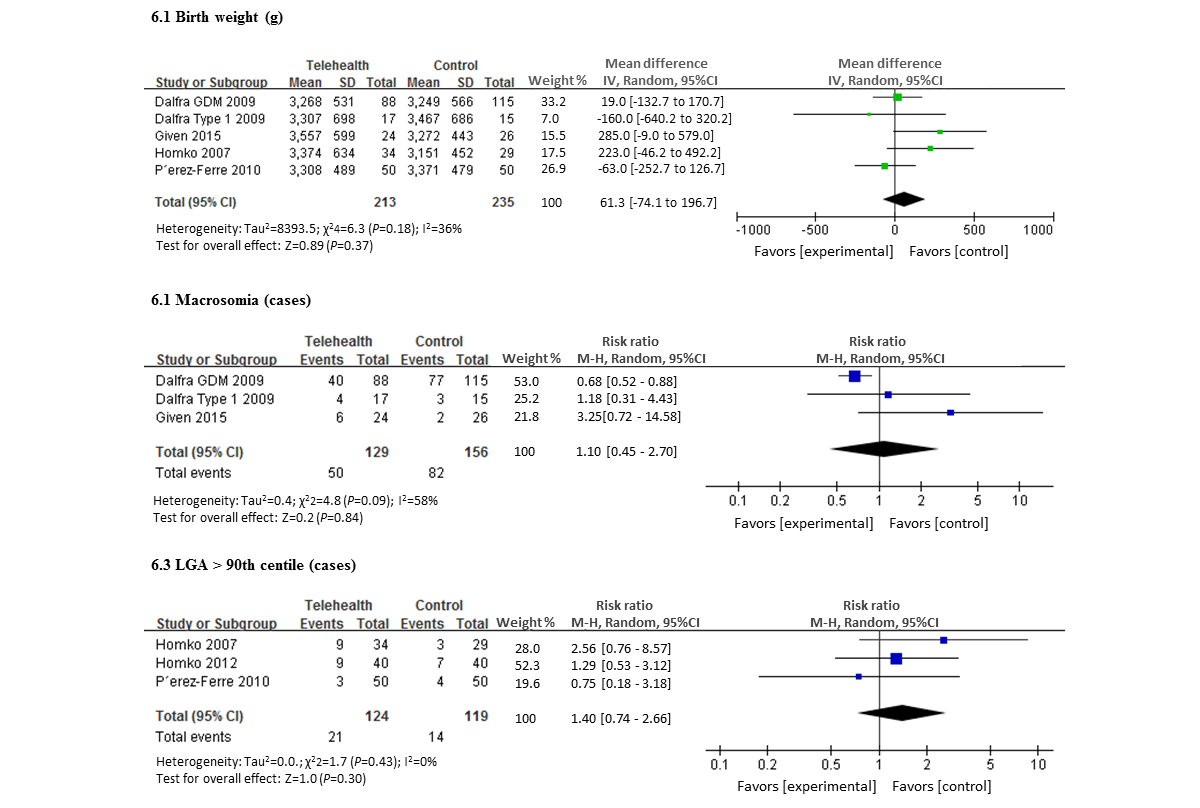
Forest plot showing the pooled clinical parameter at birth (telemedicine vs control group).

There was no significant difference between the groups with respect to mean birth weight. For the telemedicine group this was 3363 g (SD 115 g) and for the standard care group it was 3302 g (SD 121 g), with the mean gestational age at delivery of 37.9 weeks (SD 1.39 and 1.70) weeks in both groups (Figure 6). In the 2 trials that reported rates of macrosomia, there was no significant difference between the 2 groups, with an overall rate of 46% (129 cases and 159 controls, including 32 type 1 diabetic women) [21]. Three trials reported LGA as an outcome (124 women using telemedicine, and 119 with standard care) [27-29]. The overall prevalence of LGA in these 3 trials was 14.4%, with no difference demonstrated between the 2 groups.

**Figure 7:**
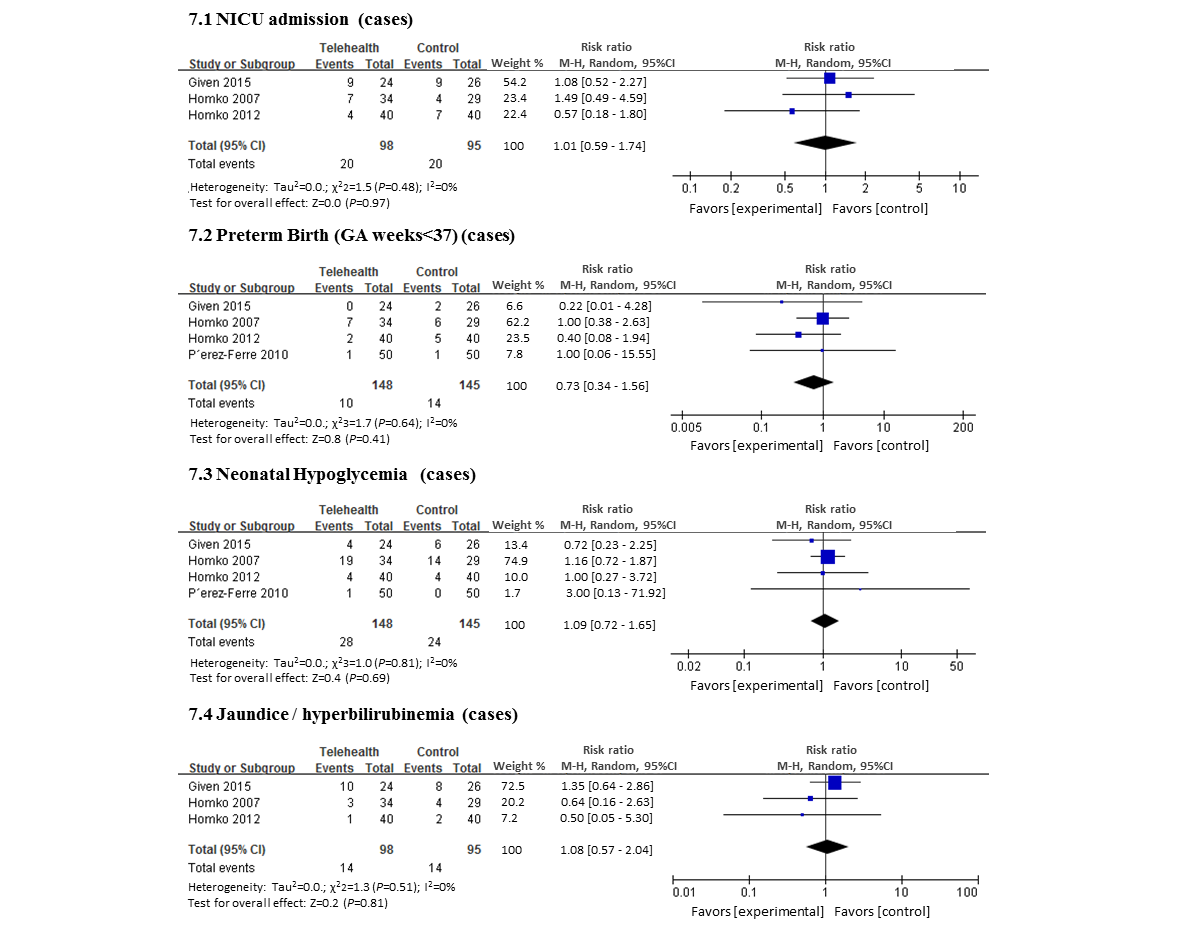
Forest plot showing the pooled risk of neonatal complication (telemedicine vs control group).

There were 40 babies of 193 (20.7%) that were admitted to the NICU, however, this proportion was not significantly different between the 2 groups (Figure 7) [27,28]. Four trials reported the proportion of babies treated for neonatal hypoglycemia [27-29]. Overall, although 18.0% (18/100) of babies were treated for hypoglycemia, there was no evidence of differences between the intervention and control groups.

### Maternal Satisfaction

One trial reported mothers' satisfaction; however, this information was only presented for the intervention group. It reported that 90% (17/19) of women in the telemedicine group agreed or strongly agreed that they were satisfied with the system and would use it again.

### Health Care Utilization

No trials provided economic or health utilization analyses. One trial described differences in the duration of clinic visits, reporting that the telemedicine visits were 8 min less than those for standard care [19].

## Discussion

### Principal Findings

While telemedicine may offer a little advantage in terms of glycemic control in pregnant women with diabetes, there is insufficient evidence at this time to support that it has any effect on other clinical endpoints. However, the 7 trials included in our meta-analysis were all small, assessed different technologies and were deemed to contain moderate to high potential sources of methodological bias. Thus, while it is reassuring that there is no evidence of harm associated with telemedicine, it is not possible to conclude whether it offers genuine benefits.

The strengths of our review were the robust and rigorous search strategy used, identifying 3 additional trials those that had been considered in previous reviews of this topic [22,32]. We included pregnant women with all forms of diabetes, as the benefits of these technologies may not be limited to women with only GDM. There are some limitations of this review. With no agreement between the trials on the screening method and definition of GDM, or standard treatment protocols, patient groups across trials may not be precisely comparable. This is a problem for all research in GDM, and unifying clinical practice was part of the motivation behind the World Health Organization (WHO) or International Association of Diabetes in Pregnancy Study Group (IADPSG) guidelines for the diagnosis of GDM. With the rapid development of advances in communication technology, the same system has not been compared in different populations, and there has been no evidence of sustained scale-up of any of these technologies. This makes it difficult to recommend any 1 system over another. Despite these differences, as the underlying concept of remotely communicating blood glucose readings between outpatient visits was the same across these trials, therefore we deemed these trials as suitable for meta-analysis. A further limitation of this review is that some of the outcomes examined, such as Cesarean section rates, gestational age at delivery, and admission to the NICU, may be more influenced by local practice, rather than being directly influenced by the intervention itself. The recent initiative by the IADPSG to attempt to standardize reporting and outcomes in diabetic pregnancy research could be a valuable advance in the future to ensure results are more comparable in this area research [33].

As stated, the sample sizes in all these trials was small. In GDM research, trials powered to detect a difference in important adverse clinical outcomes generally need to recruit around 1000 women [34,35]. Even with meta-analysis therefore, this analysis is likely to be underpowered to detect any effect on severe less common perinatal outcomes, such as shoulder dystocia and death.

Two earlier reviews on telemedicine in the management of the pregnancy with GDM have previously been published [22,32]. Mastrogiannis et al presented a narrative synthesis of trials published on telemedicine for diabetic pregnancies published before 2012. The authors concluded that telemedicine solutions for pregnant women with diabetes could reduce patient visits and potentially improve quality of life, without increasing the risk of the maternal and neonatal outcomes. Rasekaba et al presented a meta-analysis limited to women with only GDM. They identified 4 publications from 1990 to 2013 and concluded that there was insufficient evidence to support clinical benefit. Other possible benefits, such as economic savings or patient satisfaction, were not assessed. Rasekaba concluded that there was a non-significant trend to better the HbA1c of the telemedicine group [22]. By identifying and including additional trials, we have been able to demonstrate that this difference is significant both for all women with any form of diabetes in pregnancy, and those with GDM only. However, this outcome should be interpreted with caution; iron deficiency and the increased turnover of red blood cells in pregnancy can make HbA1c a less sensitive indicator of glycemic control in pregnancy [36]. Similar to our findings, Rasekaba et al did not find any difference in other clinical outcomes [22].

Whereas there were no randomized trials that assessed maternal satisfaction, there is evidence from nonrandomized trials that telemedicine is associated with high levels of satisfaction. [24] Women report these systems to be convenient to use, particularly if they live far from the hospital, have other caring responsibilities, or need to take time off work to attend appointments [1,24,37]. These observations have only been assessed in women with GDM, and ideally should be confirmed for women with type 1 and 2 diabetes among whom a reduction in clinic visits may not be desirable, however greater supervision and support may be associated with benefits in itself.

There is limited evidence that fewer outpatient visits may be needed for women with GDM using telemedicine systems [38]. We did not identify any formal health economic evaluations of telemedicine systems for gestational diabetes. In nondiabetic pregnant women, an economic analysis was conducted for a telemonitoring system designed for high-risk pregnant women in the Netherlands [39]. The system evaluated involved self-measurement and transmission of blood pressure, temperature, cardiotocography (CTG), and weight and urine albumin to a clinical care provider. This system demonstrated a cost-benefit system when compared with in-patient care. However, as this system did not measure blood glucose, and as admission for blood glucose monitoring is rare in developed countries, results cannot be extrapolated to the diabetic pregnant population. In the nonpregnant population 1 meta-analysis has assessed the economic impact of telemedicine for adults with type 2 diabetes [39,40]. The authors identified 2 papers that assessed cost-effectiveness. However, owing to small numbers and lack of consistency in the reporting of costs and outcomes, no conclusion could be drawn. A comprehensive cost analysis of direct and indirect costs is ideally needed before widespread adoption of these systems into clinical care [41].

### Conclusions

There is insufficient evidence to conclude that for women with diabetes in pregnancy, telemedicine systems produce superior clinical outcomes when compared with standard care. The reasons for this may be due to the existing studies being underpowered to detect small effect sizes and heterogeneity in the available technologies and methods by which they have been assessed. It may be however that the main benefits of these technologies are in improving maternal satisfaction and streamlining clinical care delivery. High-quality research is still needed to determine the efficacy, satisfaction, burden to pregnant women and to the health care system, and economic impact of telemedicine systems for this patient group.

## Appendix

Multimedia Appendix 1 Study characteristics.

## Abbreviations

CTG: cardiotocography
GDM: gestational diabetes mellitus
HbA1c: hemoglobin A1c
LGA: large for gestational age
NICU: neonatal intensive care unit
PRISMA: Preferred Reporting Items for Systematic Reviews and Meta-Analyses
RCT: randomized controlled trials
SBGM: self-blood glucose monitoring
SD: standard deviation
