# Supplementary material for: Telemedicine Technologies for Diabetes in Pregnancy: A Systematic Review and Meta-Analysis
Source: J Med Internet Res. 2016 Nov 9;18(11):e290. doi: 10.2196/jmir.6556 (PMC5121530; doi:10.2196/jmir.6556)
Supplement: Multimedia Appendix 1 [file jmir_v18i11e290_app1.pdf]

Table 2. Study characteristics.

Study (Year)	Area	Randomization method	Type of Diabetes Mellitus	Number of subjects (I:C)	Technology assessed (intervention)	Comparison group	Clinical parameters				Satisfaction
							Glucose level	HbA1c	Maternal	Neonatal	
Fallucca (1996) [15]	Italy	Stratified block randomization	Type 1	19 (9:10)	*Modem* transmission of BG[c] control with physician advice. Clinic visit every 2-4 weeks	Routine care, weekly visits			✓		
Wojcicki (2001) [31]	Poland	not described	Type 1	32 (17:15)	Transmission of stored BG results and information about insulin, diet over *modem* to a central hospital computer, with diabetologist review and phone call is needed to the patient.	Routine care, visits every 3 weeks	✓	✓	✓		
Homko (2007) [29]	USA	not described	GDM[a]	63 (34:29)	Provision of computers and connection to a *website* to document glucose values, transmit them at least 3 times per week, and promote communication between the patient and diabetes team	Routine care, Trial log books	✓	✓	✓	✓	
Dalfra	Italy	sequential	Type 1	32	Transmission of BG results via an	Routine care,		✓	✓	✓	

(Type 1; 2009) [21]		allocation		(17:15)	audio tone transmitted down normal *telephone receiver*. Physicians reviewed the results and transferred messages via the same system with SMS alerts when a new message was available. Clinic visits once a month	fortnightly visits					
Dalfra (GDM; 2009) [21]	Italy	sequential allocation	GDM[a]	203 (88:115)	Transmission of BG results via an audio tone transmitted down normal *telephone receiver*. Physicians reviewed the results and transferred messages via the same system with SMS alerts when a new message was available. Clinic visits once a month	Routine care, fortnightly visits		✓	✓	✓	
Perez-Ferre (2010) [30]	Spain	not stated	GDM[a]	100 (50:50)	*Cellular phone transmission* of BG results via SMS sent once a week. Results reviewed on a web based program by health professionals and text messages sent.	Routine care, monthly visits		✓	✓	✓	
Homko (2012) [28]	USA	adequate allocation concealment methods	GDM[a]	80 (40:40)	*Internet and telephone transmission* of BG data and messages from patients. Nurses check the results and send text messages to patients. At	Routine care, trial log book, fortnightly	✓		✓	✓	

					least weekly transmission.	visits					
Given (2015) [27]	Ireland	Stratified block, computer generated randomization	GDM[b]	50 (24:26)	Weekly review using telemedicine. Use of a *telemedicine hub installed in the patient's home* that would send the last 7 days of readings to a central server where accessed by a health care provider. The staff contacted the patients if changes were needed.	Routine care, fortnightly visits		✓	✓	✓	✓

[a]Carpenter and Coustan guideline.

[b]NICE 2008 guideline.

[c]BG: blood glucose.
